# Supplementary material for: Sexual Violence, Disclosure Pattern, and Abortion and Post-Abortion Care Services in Displaced People’s Camps in Africa: A Scoping Review
Source: Int J Environ Res Public Health. 2024 Jul 30;21(8):1001. doi: 10.3390/ijerph21081001 (PMC11353391; doi:10.3390/ijerph21081001)
Supplement: Supplementary file 1 [file ijerph-21-01001-s001.zip › ijerph-2993622-supplementary.pdf]

Supplementary Materials

File S1. Ethical Consideration Certificate

Covenant University

Canaanland, Km 10, Idiroko Road, Ota, Ogun State, Nigeria.
Tel:+234-09033550046
Website: www.covenantuniversity.edu.ng

THE WORLD UNIVERSITY RANKINGS 2022 TOP 800

CHAIRMAN, COVENANT HEALTH RESEARCH ETHICS COMMITTEE (CHREC)
Email: hrec@covenantuniversity.edu.ng
COVENANT UNIVERSITY P.M.B. 1023, OTA, OGUN STATE, NIGERIA

Our Ref: CU/HREC/PA/164/22 **Date 14th October, 2022Your Ref:**
NOTICE OF APPROVAL OF PROTOCOL AFTER FULL COMMITTEE REVIEW

RE: SEXUAL VIOLENCE, DISCLOSURE PATTERN, AND ABORTION AND POST ABORTION CARE SERVICES IN DISPLACED PEOPLES' CAMPUS IN AFRICA: A SCOPING REVIEW

**US DEPT. OF HEALTH & HUMAN SERVICES NUMBER**	**IORG0010037 CHREC NHREC REG. NHREC/25/10/2021**
**HREC Protocol Assigned Number**	**CHREC /162/2022**
**Name of Principal Investigator**	**Dr. Paul Adekola**
**Date of Receipt of Valid Application:**	**8th October, 2022**
**Date of Meeting where decision was taken**	**14th October, 2022**
**The approval dates from**	**14th October, 2022 to 13th October, 2023**

We write to inform you that the research described in your submitted protocol and other related documents has undergone a positive review and given approval following the outcome of the review by the Covenant Health Research Ethics Committee (CHREC).

In a multi-year research, endeavor to submit your annual report to the CHREC early in order to obtain renewalof your approval and avoid disruption of your research.

The national Code for Health Research Ethics requires you to comply with all institutional guidelines, rules and regulations and with the tenets of the Code including ensuring that all adverse events are reported promptlyto the CHREC. No changes are permitted in the research without prior approval by the CHREC except in circumstances outlined in the Code. The CHREC reserves the right to conduct compliance visit to your research site without previous notification.

Prof. Grace I. Olasehinde
Chairman, CHREC

Prof. Solomon O. Rotimi
Secretary, CHREC

Raising A New Generation of Leaders

Vice-Chancellor:
Professor Abiodun H. Adebayo

Ag. Registrar:
Mr. Emmanuel Igban
